# Phosphorylation of Notch1 by Pim kinases promotes oncogenic signaling in breast and prostate cancer cells

**DOI:** 10.18632/oncotarget.9215

**Published:** 2016-05-07

**Authors:** Niina M. Santio, Sebastian K.-J. Landor, Laura Vahtera, Jani Ylä-Pelto, Elina Paloniemi, Susumu Y. Imanishi, Garry Corthals, Markku Varjosalo, Ganesh Babu Manoharan, Asko Uri, Urban Lendahl, Cecilia Sahlgren, Päivi J. Koskinen

**Affiliations:** ^1^ Section of Genetics and Physiology, Department of Biology, University of Turku, Turku, Finland; ^2^ Drug Research Doctoral Programme, University of Turku, Turku, Finland; ^3^ Turku Centre for Biotechnology, University of Turku and Åbo Akademi University, Turku, Finland; ^4^ Department of Cell and Molecular Biology, Karolinska Institutet, Stockholm, Sweden; ^5^ Turku University of Applied Sciences, Turku, Finland; ^6^ Institute of Biotechnology, University of Helsinki, Helsinki, Finland; ^7^ Institute of Chemistry, University of Tartu, Tartu, Estonia; ^8^ Department of Biomedical Engineering, Institute for Complex Molecular Systems, Eindhoven University of Technology, Eindhoven, The Netherlands; ^9^ Current address: Faculty of Pharmacy, Meijo University, Nagoya, Japan; ^10^ Current address: Van't Hoff Institute for Molecular Sciences, University of Amsterdam, Amsterdam, The Netherlands

**Keywords:** Notch1, Pim kinases, migration, metabolism, tumorigenesis

## Abstract

Tumorigenesis is a multistep process involving co-operation between several deregulated oncoproteins. In this study, we unravel previously unrecognized interactions and crosstalk between Pim kinases and the Notch signaling pathway, with implications for both breast and prostate cancer. We identify Notch1 and Notch3, but not Notch2, as novel Pim substrates and demonstrate that for Notch1, the serine residue 2152 is phosphorylated by all three Pim family kinases. This target site is located in the second nuclear localization sequence (NLS) of the Notch1 intracellular domain (N1ICD), and is shown to be important for both nuclear localization and transcriptional activity of N1ICD. Phosphorylation-dependent stimulation of Notch1 signaling promotes migration of prostate cancer cells, balances glucose metabolism in breast cancer cells, and supports *in vivo* growth of both types of cancer cells on chick embryo chorioallantoic membranes. Furthermore, Pim-induced growth of orthotopic prostate xenografts in mice is associated with enhanced nuclear Notch1 activity. Finally, simultaneous inhibition of Pim and Notch abrogates the cellular responses more efficiently than individual treatments, opening up new vistas for combinatorial cancer therapy.

## INTRODUCTION

Notch signaling is frequently deregulated in aggressive forms of both hematopoietic malignancies and solid tumors [1–4]. High Notch1 levels have been linked to poor prognosis in breast cancer [5], where Notch1 has been shown to induce epithelial-to-mesenchymal transition (EMT) [6, 7], upregulate extracellular matrix metalloproteinases [8], and induce a switch to glycolytic metabolism [9], contributing to both tumor initiation and progression. Similarly in prostate cancer, Notch1 supports cancer cell survival and EMT [10]. While deregulated Notch signaling is often involved in metastatic growth as well as therapy resistance, there are also contradictory data, suggesting that the oncogenic effects of Notch are highly dependent on the cellular context [11].

Although the molecular components of Notch signaling have been well defined, the mechanisms regulating Notch activity have not yet been fully characterized [12]. The Notch intracellular domain (NICD) represents the active moiety of the receptor and is generated via proteolytic cleavage of the full-length transmembrane protein. Diversity in cellular responses is created by the four different Notch family receptors (Notch1-4), their five different ligands (Jagged1, 2; Delta-like1, 3, 4) [1, 12], as well as by the DNA-binding transactivator CSL/RBP-Jκ, which exhibits differential binding preferences to the four NICDs [13–15]. Signaling activity is regulated by the intersection of Notch with other signaling pathways, including BMP/TGF-β, Wnt, PI-3 kinase and the cellular hypoxic response [9, 12]. Direct fine-tuning at the molecular level is mediated via post-translational modifications of Notch receptors and ligands. The Notch receptors can be modified by ubiquitylation, sumoylation, hydroxylation, acetylation and phosphorylation, all of which can specify the signaling output [12]. Even though Notch family members are phosphoproteins, limited information is available concerning the kinases involved or the physiological relevance of their phosphorylation. Phosphorylation by the cyclin-dependent kinase 8 targets NICD for proteasomal degradation [16], while the atypical protein kinase Cζ regulates Notch1 endocytosis and activity [17], but additional Notch kinases are likely to exist.

Analogous to Notch proteins, oncogenic Pim family kinases (Pim1-3) contribute to development of both hematopoietic malignancies and solid tumors [18–21] by supporting cell survival [22, 23], promoting cancer cell migration and invasion [24–26], and controlling mitochondrial integrity as well as glucose metabolism [27, 28]. Via phosphorylation, the serine/threonine-specific Pim kinases positively or negatively regulate activities of several cellular or viral transcription factors, including nuclear antigens of tumorigenic herpesviruses [29, 30]. Interestingly, these viral factors do not directly bind to DNA, but control gene expression by hijacking the transcriptional machinery of their host cells and by interacting with the Notch coactivator CSL [31], raising the question of whether Pim kinases can also directly or indirectly modulate Notch signaling.

In this report, we demonstrate that Pim kinases phosphorylate Notch1 on Serine 2152 within the intracellular domain, and thereby enhance the nuclear localization and activity of Notch1. This crosstalk between Pim and Notch proteins enhances tumorigenic growth of breast and prostate cancer cells via cell type-specific effects, by balancing breast cancer cell metabolism and by promoting prostate cancer cell motility, respectively.

## RESULTS

### Pim kinases upregulate endogenous Notch activity

**Figure 1:**
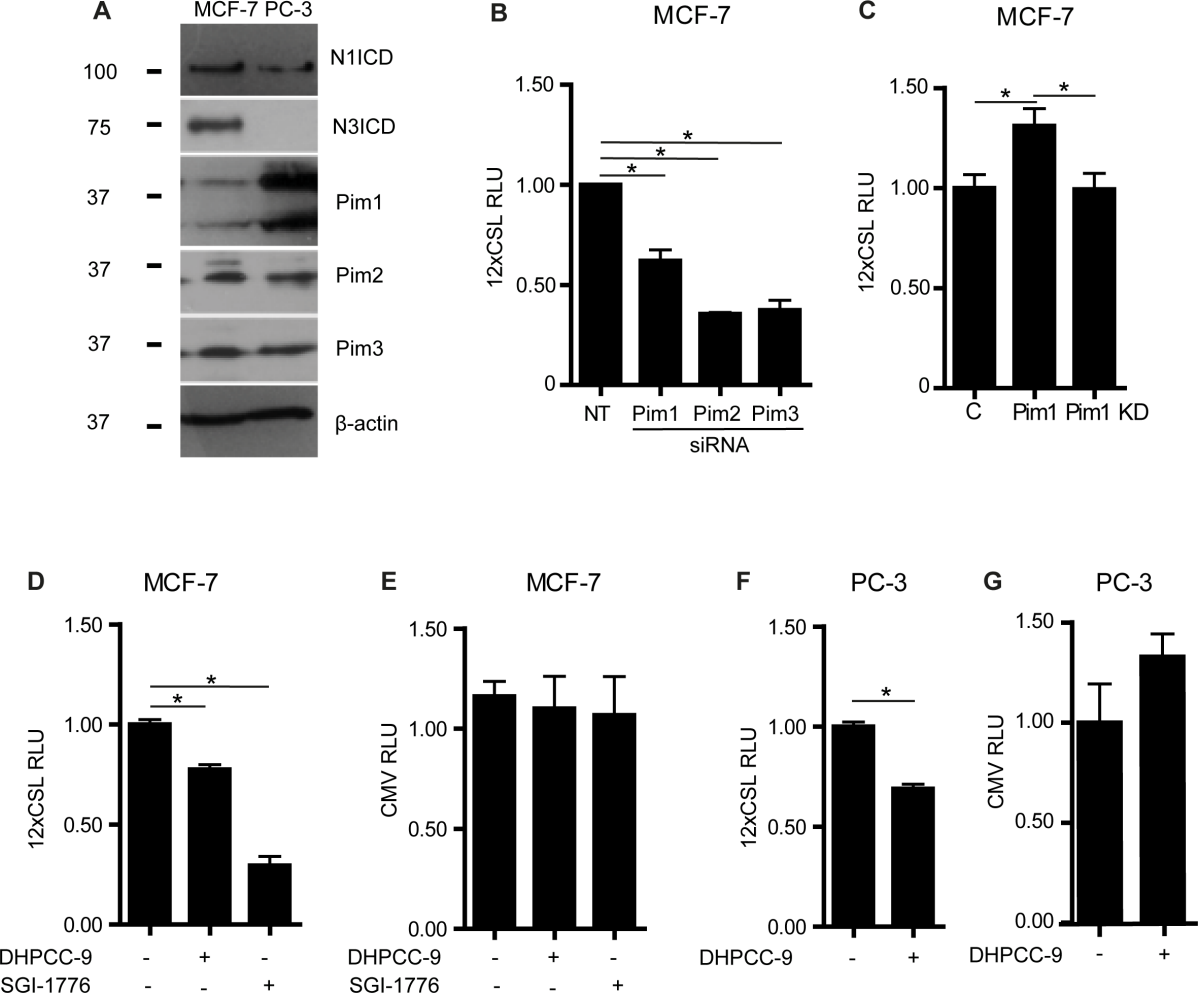
Pim kinases enhance endogenous Notch activity **A.** Endogenous expression levels for intracellular domains of Notch1 (N1ICD) or Notch3 (N3ICD), and for Pim family kinases were analysed by Western blotting from MCF-7 and PC-3 cell lysates. β-actin levels were used as loading controls. **B.** Endogenous Notch activity was measured by CSL-dependent luciferase reporter assays in MCF-7 transiently transfected with non-targeting (NT) or Pim gene-specific siRNAs. RLU, relative light unit. **C.** Similar assays were carried out in cells transiently transfected with an empty control vector (C), wild-type (WT) or kinase-deficient (KD) Pim1. **D-G.** CSL- or CMV-dependent luciferase reporter assays were performed with untransfected MCF-7 or PC-3 cells that had been treated for 24 h with 0.1% DMSO (−), 10 μM DHPCC-9 or 10 μM SGI-1776. Shown are representative graphs from three independent luciferase experiments with average data from three parallel samples.

To assess the putative effects of Pim kinases on Notch activity, we used cancer cell lines, which endogenously express both Pim and Notch family members. Western blot analyses showed that Notch1 and Notch3 as well as all three Pim family kinases are expressed in MCF-7 breast cancer cells (Figure 1A), whereas PC-3 prostate cancer cells express all but Notch3. Endogenous Notch activity was then measured by CSL-dependent luciferase reporter assays from MCF-7 cells, which had been transfected with previously validated reagents [24] to decrease or increase Pim expression or activity. RNA interference oligonucleotides targeting individual Pim family members reduced reporter activity, while ectopic overexpression of Pim1 enhanced it (Figure 1B-1C). The kinase activity of Pim1 was essential for its enhancing effects, since a kinase-deficient (KD) mutant of Pim1 remained ineffective (Figure 1C). In addition, Notch reporter activity was reduced by two structurally unrelated Pim-selective inhibitors, DHPCC-9 and SGI-1776 (Figure 1D). Reporter activity was inhibited also in PC-3 cells treated with DHPCC-9 (Figure 1F), while the constitutive activity of a CMV-driven control reporter was not decreased by Pim inhibitors in either cell line (Figure 1E, 1G). These results confirm that the negative effects of the inhibitors on the CSL-dependent reporter activity are specific and not simply due to cytotoxicity. In sum, these data indicate that Pim kinase expression and activity enhance endogenous Notch activity.

To further explore the physiological relevance of the observed crosstalk between Pim and Notch in cancer, we searched for correlations between their mRNA levels in primary breast and prostate cancer samples using the MediSapiens database. These analyses revealed positive Pearson correlations between *PIM1* and *NOTCH1* in both types of cancer as well as between *PIM1* and *NOTCH3* in breast cancer (Supplementary Figure 1A-1B). By contrast, no correlations were found between *PIM1* and *NOTCH3* in prostate cancer or between *PIM1* and *NOTCH2* in breast cancer (Supplementary Figure 1A-1B).

### Pim kinases phosphorylate Notch1 at serine 2152 in the intracellular domain

**Figure 2:**
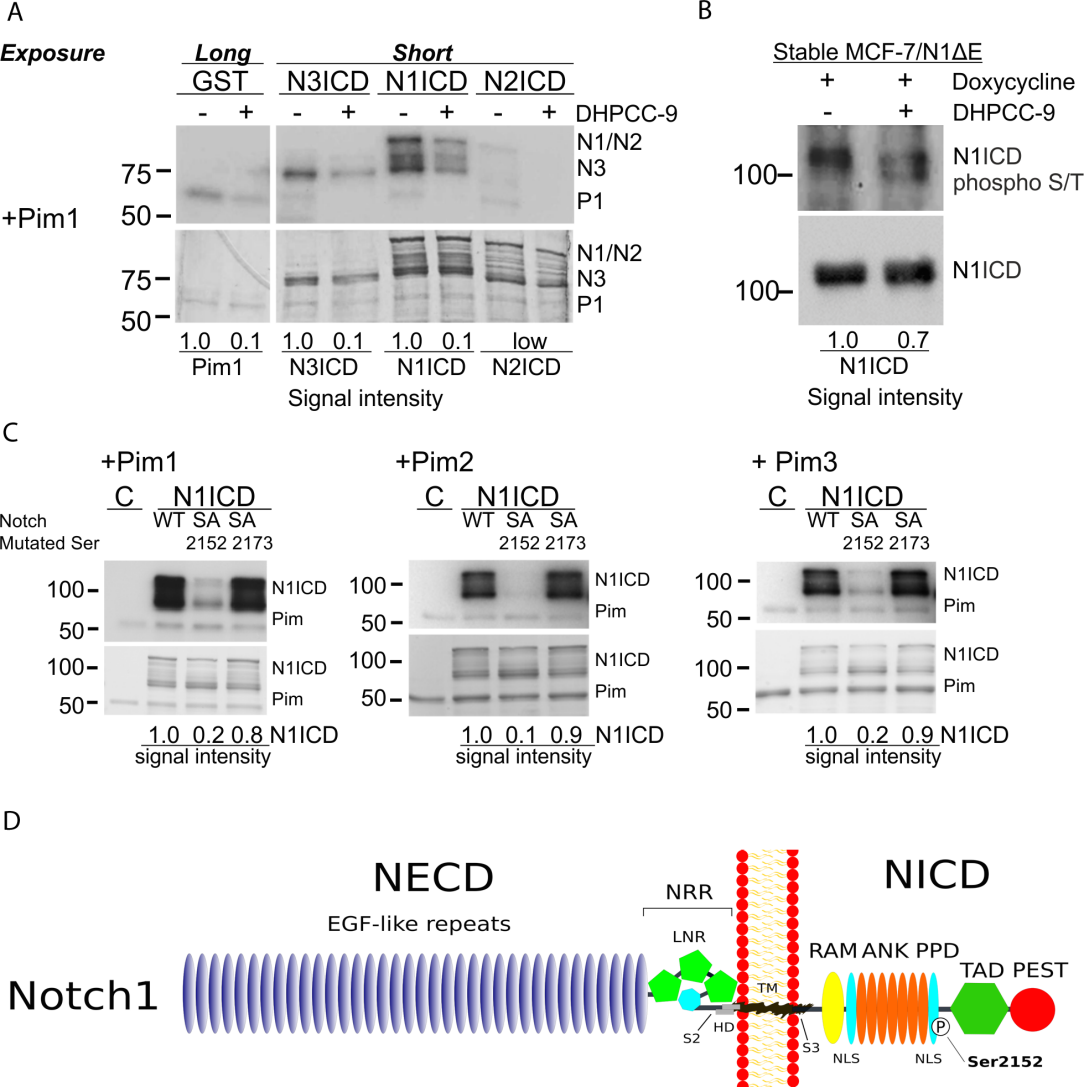
Serine 2152 in Notch1 is phosphorylated by Pim kinases **A.** GST-tagged Pim kinases were treated with 0.1% DMSO or 10 μM DHPCC-9 prior to *in vitro* kinase assays with GST-tagged NICDs or GST control protein. Pim (P) autophosphorylation and NICD (N) phosphorylation signals were analysed by autoradiography (above), while protein loading was detected by Page Blue™ staining (below). **B.** N1ICD was immunoprecipitated from MCF-7 cells that stably expressed the doxycycline-inducible N1ΔE protein and that were treated with 10 μM DHPCC-9 and/or 1 μg/ml of doxycycline for 24 h, after which the phosphorylation status of N1ICD was analysed by Western blotting with antibodies targeting phosphorylated S/T residues or N1ICD. **C.** Phosphorylation of wild-type (WT) N1ICD or phosphodeficient (SA) mutants by Pim family members were analysed by *in vitro* kinase assays. At least two independent experiments were performed and shown are representative results of autoradiography (above) and protein staining (below) in one experiment. **D.** A schematic model shows Pim target sites within the Notch1 protein. Abbreviations: NECD = The Notch extracellular domain, EGF = Epidermal Growth Factor, NRR = negative regulatory region, LNR = the Lin12-Notch repeat, HD = heterodimerization domain, S2 = ADAM family metalloprotease cleavage site, TM = the transmembrane domain, S3 = γ-secretase cleavage site, RAM = Rbp-associated molecule domain, ANK = ankyrin repeat domain, PPD = potential phosphorylated domain, NLS = nuclear localization signal, TAD = transcription activation domain, PEST = domain rich in proline, glutamic acid, serine and threonine.

Since Pim kinases increased and Pim inhibition reduced Notch activity, we next addressed whether Pim kinases directly target Notch ICDs. Glutathione S-transferase (GST)–tagged NICDs were subjected to *in vitro* kinase assays with GST-Pim1. Interestingly, Pim1 phosphorylated Notch1 and Notch3, but not Notch2 ICD (Figure 2A), which was in line with the observed Pearson correlations (Supplementary Figure 1). As expected, DHPCC-9 treatment reduced Pim1-mediated phosphorylation (Figure 2A), while the inactivating mutation in Pim1 KD completely abolished it (Supplementary Figure 2A).

To verify that Pim kinases can phosphorylate Notch1 in cells, we used a stable MCF-7/N1ΔE cell line, where a membrane-tethered, ligand-independent form of Notch1 (N1ΔE) is expressed in a doxycycline-inducible fashion and processed by the endogenous γ-secretase to generate N1ICD. MCF-7/N1ΔE cells were treated with doxycycline and DMSO or DHPCC-9, after which N1ICD was immunoprecipitated and its phosphorylation status analysed by Western blotting using an antibody recognizing serine or threonine residues phosphorylated by basophilic kinases. DHPCC-9 treatment reduced phosphorylation of N1ICD and thereby also increased its gel migration (Figure 2B).

Using mass spectrometry, we identified the serine residue 2152 as the major Pim1 target site in Notch1 (Supplementary Figure 2B-2C). The amino acid sequence around S2152 (K-A-R-K-P-S-T) shares high complementarity with the Pim1 consensus sequence K/R-K/R-R-K/R-X-S/T-X′, where X′ is defined as an amino acid with neither a basic nor a large hydrophobic residue chain [32]. However, *in silico* analysis suggested another putative site at S2173 with a similar complementarity to Pim target sequence (A-R-R-K-K-S-Q). Therefore, site-directed mutagenesis was used to replace either S2152 or S2173 with an alanine residue to generate phosphodeficient mutants. Results from *in vitro* kinase assays revealed that S2152, but not S2173 in N1ICD is phosphorylated by all three Pim kinases (Figure 2C). Serine 2152 is localized in the N1ICD within a potential phosphorylated domain (PPD) at the second nuclear localization signal (NLS) (Figure 2D). When a sequence comparison between Notch family members was performed, mouse and human Notch1 showed high complementarity at the amino acid sequence around S2152 (Supplementary Table 1). For further analyses, we generated a phosphomimicking mutant, where the serine residue was replaced with glutamic acid. From here on, the phosphodeficient mutant is denoted as SA (Notch1 S2152A) and the phosphomimicking mutant as SE (Notch1 S2152E).

### Phosphorylation at Pim target sites increases Notch1 nuclear localization and activity

**Figure 3:**
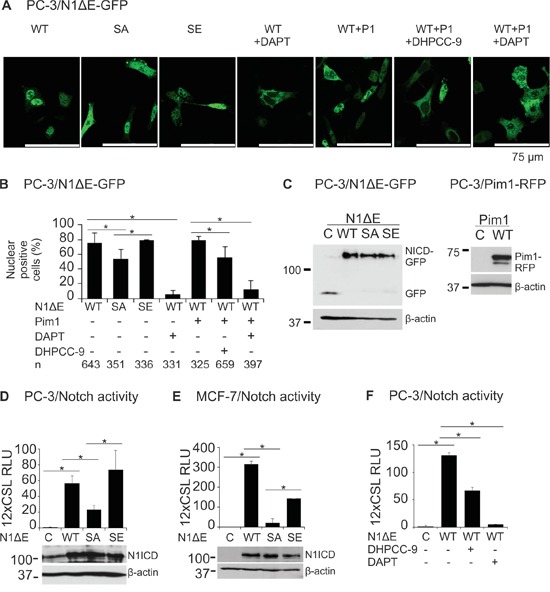
Phosphorylation enhances both nuclear localization and activity of Notch1 **A.** PC-3 cells were transiently transfected with GFP-tagged wild-type (WT), phosphodeficient (SA) or phosphomimicking (SE) N1ΔE. N1ICD localization was imaged by confocal microscopy also from cells co-transfected with the RFP-tagged Pim1 (P1) and/or treated for 24 h with 0.1% DMSO, 5 μg/ml of DAPT or 10 μM DHPCC-9. **B.** Shown is the average nuclear localization of N1ICD, as determined from two independent experiments along with the analysed cell numbers (n). **C.** Equivalent expression levels for the GFP- or RFP-tagged wild-type or mutant proteins were confirmed by Western blotting. **D.** CSL-dependent luciferase reporter assays were used to detect Notch activity in PC-3 cells transiently overexpressing untagged wild-type or phosphomutant N1ΔE proteins, the equivalent expression levels of which were confirmed by Western blotting. **E.** Notch wild-type and phosphomutant activities were similarly measured in MCF-7 cells. **F.** The effects of DHPCC-9 or DAPT treatments on the activity of wild-type Notch1 protein were measured in PC-3 cells. Reporter assays were repeated at least three times and shown are average results from one or more independent experiments.

To explore the functional consequences of Pim-mediated phosphorylation of Notch1, we generated constructs expressing RFP-tagged Pim1 and GFP-tagged Notch1ΔE wild-type or phosphomutant proteins and transiently overexpressed combinations of them in PC-3 cells. When we analysed the localization of GFP-tagged proteins in these cells, both the wild-type Notch1 protein and the SE mutant mainly localized in the nuclei, while significantly fewer GFP-positive nuclei were observed in cells expressing the SA mutant (Figure 3A-3B). Treatment with the Pim inhibitor DHPCC-9 similarly decreased the presence of wild-type Notch1 in the nuclei, while the γ-secretase inhibitor DAPT completely blocked the cleavage-dependent nuclear translocation of Notch1. Furthermore, the effects of DAPT could not be rescued by coexpressed Pim1. Western blotting assays verified the expression levels for fluorescent Notch1 and Pim1 proteins (Figure 3C).

Supporting results were obtained with CSL-dependent luciferase reporter assays with untagged Notch1 proteins. Ectopic expression of either wild-type N1ΔE or the SE mutant resulted in vastly elevated luciferase levels as compared to untransfected PC-3 or MCF-7 cells (Figure 3D-3E). By contrast, the transcriptional activity of the SA mutant was remarkably reduced. In both types of cells, treatments with either DHPCC-9 or DAPT efficiently reduced Notch1-induced reporter activity (Figure 3F and data not shown). Collectively, these data suggest that Pim-mediated phosphorylation enhances both the nuclear localization and transcriptional activity of Notch1.

### Pim1 colocalizes and interacts with Notch1

**Figure 4:**
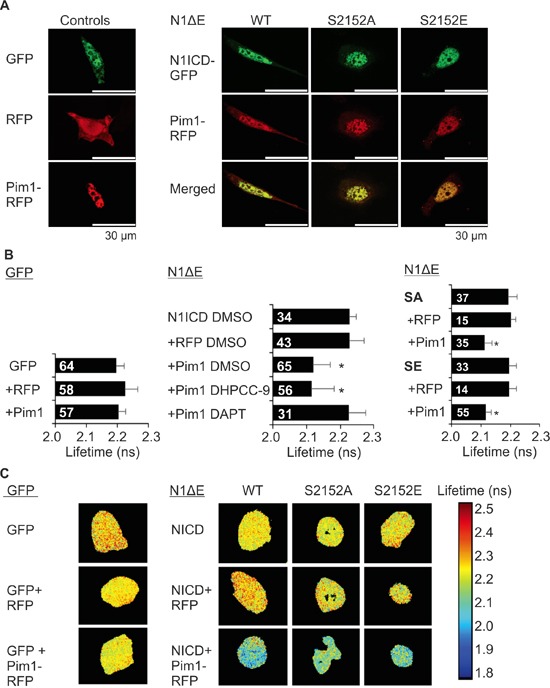
Notch1 colocalizes and interacts with Pim1 **A.** PC-3 cells were transiently transfected with GFP-tagged wild type (WT), phosphodeficient (SA) or phosphomimicking (SE) N1ICD, RFP-tagged Pim1 or empty GFP or RFP vectors. 24 h after transfection, cells were treated overnight with 0.1% DMSO, 10 μM DHPCC-9 or 5 μg/ml of DAPT. Fixed samples were analysed by confocal microscopy. Shown are representative single channel or merged images of DMSO-treated samples. **B.** Physical interactions between Pim1 and N1ICD were measured by fluorescence-lifetime imaging microscopy (FLIM). Samples expressing only GFP or GFP-tagged N1ICD were used as negative controls. Shown are average GFP lifetimes from two independent experiments along with analysed cell numbers inside the bars. **C.** Representative images from FLIM analyses.

We next assessed the physical interactions between fluorescently tagged Pim1 and N1ICD by confocal and fluorescence-lifetime imaging microscopy (FLIM). Confocal microscopy revealed that Pim1 colocalized with both wild-type and phosphomutant N1ICD within the nuclei in PC-3 cells (Figure 4A). Furthermore, significantly reduced GFP lifetimes were observed by FLIM when Pim1 and N1ICD were co-expressed, indicating that these proteins physically interact with each other (Figure 4B-4C). By contrast, this interaction was lost, when cells were treated with DAPT to prevent the cleavage and nuclear translocation of N1ICD (Figure 4B).

**Figure 5:**
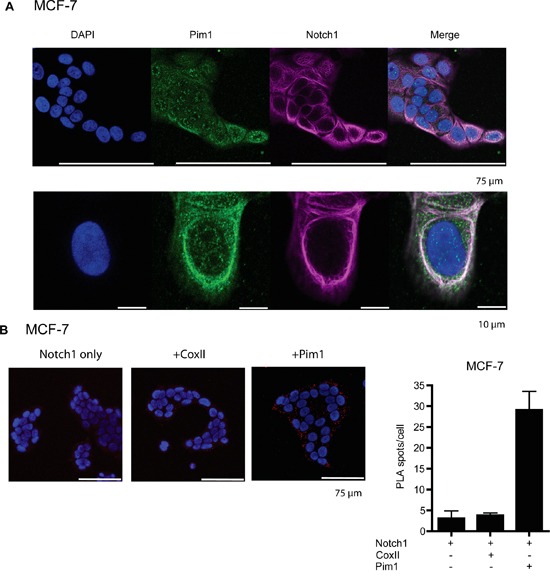
Endogenous Pim1 and Notch1 proteins interact with each other **A.** To visualize localization of endogenously expressed proteins, MCF-7 cell samples were stained with antibodies that recognized either Pim1 or both full-length and cleaved Notch1. **B.** *In situ* proximity ligation assay (PLA) was used to demonstrate physical interaction between Pim1 and Notch1. Notch1 alone or in combination with cytochrome oxidase II (CoxII) were used as negative controls.

Additional fluorescent assays were carried out to confirm that also endogenously expressed Pim1 and Notch1 proteins are able to colocalize and interact with each other. Staining of MCF-7 cells with Pim1-specific antibodies revealed that endogenous Pim1 is distributed throughout the cells in both the nucleus and the cytoplasm, but not in the nucleoli (Figure 5A). By contrast, staining with Notch antibodies that recognize both membrane-bound and cleaved Notch1 indicated that in non-stimulated MCF-7 cells, endogenous Notch1 mostly resides on the plasma membrane (Figure 5A). Thus, there was only a small fraction of N1ICD that was able to enter the nucleus and activate transcription there, as was also evident from our luciferase results (Figures 1 and 3).

The endogenous interactions of Pim1 and Notch1 were confirmed from MCF-7 cell samples by *in situ* proximity ligation assays (PLA; Figure 5B). Strong cytoplasmic signals were observed between Pim1 and Notch1, but not by Notch1 alone or in combination with cytochrome oxidase II (CoxII), which were used as negative controls. The cytoplasmic colocalization and interaction patterns of endogenously expressed Pim1 and Notch1 suggest that Pim1 phosphorylates Notch1 already in the cytoplasm and thereby promotes its nuclear translocation, as supported by the data with ectopically expressed proteins.

### Pim protein levels are upregulated by Notch1

**Figure 6:**
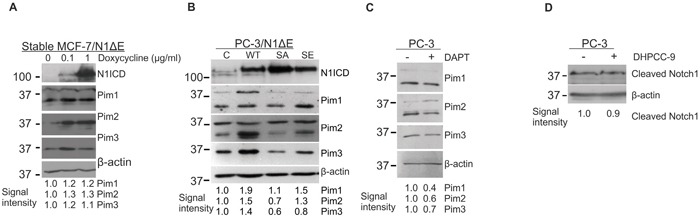
Notch1 upregulates Pim protein levels **A.** The stably transfected MCF-7/N1ΔE cells were treated with increasing amounts of doxycycline to induce Notch1 overexpression, after which N1ICD and Pim protein levels were measured by Western blotting. **B.** PC-3 cells were transiently transfected with wild-type (WT), phosphodeficient (SA) or phosphomimicking (SE) N1ΔE, and 48 hours later, the N1ICD and Pim protein levels were analysed by Western blotting. **C.** Untransfected PC-3 cells were treated with 5 μg/ml of DAPT for 24 h prior to Western blotting with Pim antibodies. **D.** Untransfected PC-3 cells were treated with 10 μM DHPCC-9 for 24 h prior to Western blotting with antibodies that specifically recognise cleaved N1ICD. Similar experiments were repeated at least twice.

To determine whether there is reciprocal regulation of Pim kinases by Notch proteins, we analysed the effects of Notch1 on Pim protein levels in MCF-7 and PC-3 cells. Since Pim kinases are constitutively active whenever expressed [33], changes in their expression levels are expected to directly correlate with their activities. Doxycycline-inducible overexpression of N1ΔE in stably transfected MCF-7 cells resulted in slightly upregulated expression of Pim family members, while more significant increases in them were observed after transient overexpression of wild-type Notch1 in PC-3 cells (Figure 6A-6B). Interestingly, the SA mutant was unable to upregulate Pim expression (Figure 6B), suggesting that phosphorylation-dependent Notch activity is necessary for the observed increase in Pim expression. Accordingly, DAPT treatment also reduced Pim protein levels in untransfected PC-3 cells (Figure 6C). By contrast, the Pim inhibitor DHPCC-9 had no effects on the endogenous expression of N1ICD, when analysed with an antibody recognizing only the cleaved form of Notch1 (Figure 6D).

### Pim-mediated cell migration is dependent on Notch1 phosphorylation and activity

**Figure 7:**
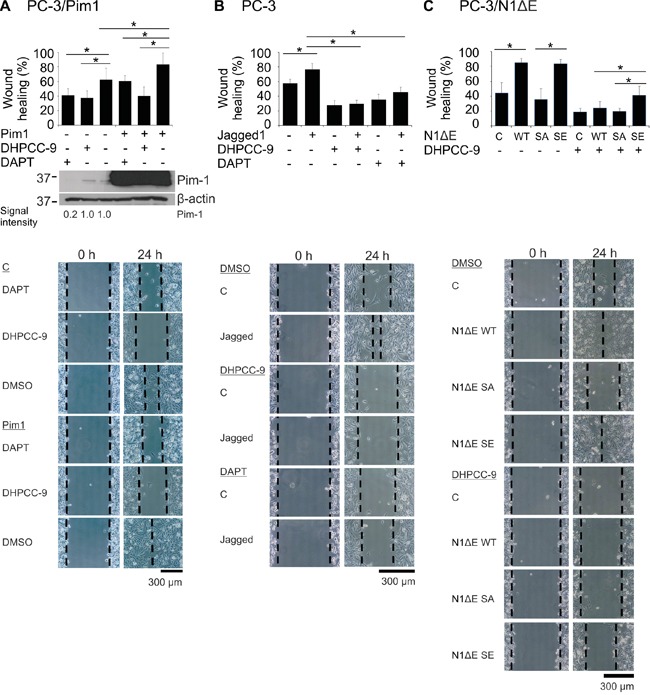
Phosphorylation of Notch1 increases PC-3 cell migration **A.** PC-3 cell migration was analysed by wound healing assays in cells transiently transfected with Pim1 and/or treated with 0.1% DMSO, 10 μM DHPCC-9 or 5 μg/ml of DAPT for 24 h. Pim expression levels from the wound healing samples were analysed by Western blotting. **B.** Similar wound healing assays were performed with PC-3 cells that had been plated onto wells coated with the Notch ligand Jagged1. **C.** N1ΔE wild-type (WT), phosphodeficient (SA) or phosphomimicking (SE) mutants were overexpressed in PC-3 cells, in which cell migration was analysed after treatments with 0.1% DMSO or 10 μM DHPCC-9. Shown are average healing percentages from two or three independent experiments.

Both Pim kinases and Notch1 have been shown to promote cancer cell migration and invasion [24, 34]. To determine whether there is a hierarchical or synergistic relationship between these proteins, we performed wound healing assays in PC-3 cells. Inhibition of endogenous Notch activity by DAPT decreased cell migration to the same extent as Pim inhibition by DHPCC-9, even when Pim1 was overexpressed (Figure 7A). Conversely, activation of endogenous Notch signaling by immobilized Jagged1 ligand enhanced migration, which was antagonized not only by DAPT, but also by the Pim inhibitor DHPCC-9 (Figure 7B). Transient overexpression of wild-type N1ΔE or the SE mutant similarly increased cell migration, whereas overexpression of the SA mutant was unable to do so (Figure 7C). Furthermore, the SE mutant was even able to partly rescue the negative effects of the Pim inhibitor DHPCC-9 on cell motility. Taken together, these data indicate that PC-3 cells need activities of both Pim kinases and Notch1 for efficient cell migration.

To confirm that the increase in PC-3 cell migration was not simply due to enhanced cell proliferation, we used IncuCyte analyses to measure cell confluency at several time-points after transfection. Indeed, during the 72 h follow-up period, it became evident that overexpressed Notch1 and Pim1 proteins reduce rather than increase the ability of cells to reach confluency (Supplementary Figure 3A-3B).

### Crosstalk between Notch1 and Pim1 regulates breast cancer cell metabolism

**Figure 8:**
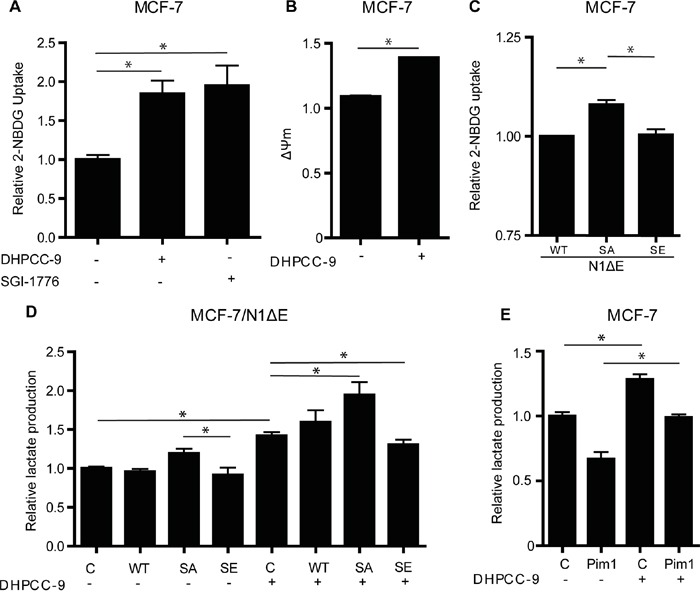
Phosphorylation of Notch1 balances MCF-7 cell metabolism **A.** Untransfected MCF-7 cells were treated overnight with 0.1% DMSO or 10 μM DHPCC-9, after which glucose uptake was measured by flow cytometry, using the fluorescent 2-NBDG probe. **B.** Mitochondrial membrane potential was similarly measured by flow cytometry, using the TMRM probe. **C.** Glucose uptake was also measured from cells transiently transfected with wild-type or mutant N1ΔE. **D.** Lactate production was measured after similar transfections and/or DHPCC-9 treatment. **E.** Lactate production was also measured after Pim1 transfection and/or DHPCC-9 treatment. Shown are average values from two to three independent experiments.

Both Pim kinases and Notch1 have been implicated in the control of glucose metabolism [9, 28]. In breast cancer cells, Notch activation induces a glycolytic switch, while Notch inhibition leads to defects in mitochondrial function and a forced glycolytic phenotype [9]. To evaluate the role of Pim kinases and their potential interplay with Notch1 in breast cancer cell metabolism, MCF-7 cells were treated with the Pim-selective inhibitors DHPCC-9 or SGI-1776. Inhibition of Pim activity increased glucose uptake in association with a higher mitochondrial membrane potential (Figure 8A-8B), which was indicative of defects in mitochondrial function. In a similar fashion, the N1ΔE SA mutant increased glucose uptake as compared to the wild-type Notch1 protein or the SE mutant (Figure 8C). When lactate production was measured as the endpoint of enforced glycolytic metabolism, both Pim inhibition by DHPCC-9 and overexpression of the N1ΔE SA mutant increased lactate levels, while the SE mutant partially rescued the effects of DHPCC-9 (Figure 8D). Conversely, overexpression of Pim1 had opposite effects (Figure 8E), supporting efficient utilization of internalized glucose for OXPHOS and protein synthesis, as previously reported [28]. Taken together, these data suggest that Pim1 counteracts the effects of Notch1 in regulation of breast cancer cell metabolism.

IncuCyte and MTT assays were performed to exclude the possibility that our results were affected by the influence of Pim or Notch upregulation or inhibition on MCF-7 cell growth rate or survival, respectively. In IncuCyte analyses, neither Notch1 nor Pim1 increased the ability of cells to reach confluency (Supplementary Figure 3C-3D). In addition, no significant differences were observed in the viabilities of cells transfected with wild-type or mutant N1ICDs or treated with DAPT or DHPCC-9 (Supplementary Figure 3E-3F). These MTT assay results are in line with our previously published data, according to which DHPCC-9 does not affect viability of PC-3 cells [24].

### Notch1 and Pim kinases synergize to promote tumor growth *in vivo*

**Figure 9:**
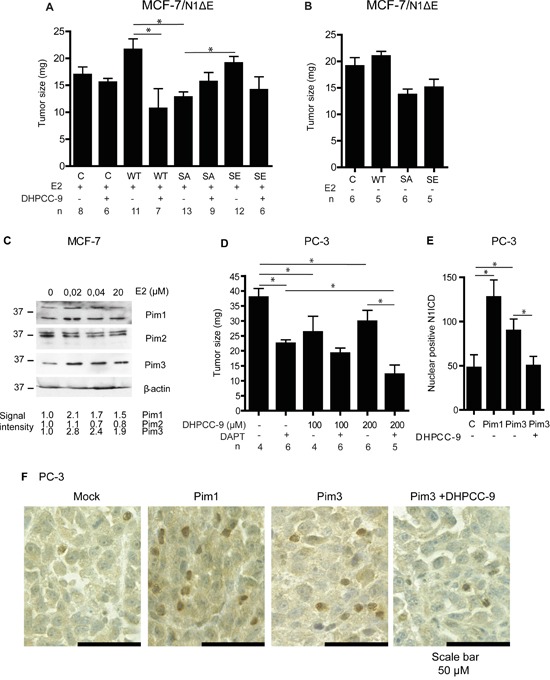
Pim1 and Notch1 synergize to promote breast and prostate xenograft growth The chorioallantoic membrane (CAM) model was used to measure tumorigenic growth of xenografted MCF-7 or PC-3 cells. **A.** MCF-7 cells transiently transfected with wild-type (WT), phosphodeficient (SA) or phosphomimicking (SE) N1ΔE were grown for 5 days on the CAM and treated daily with 30 μl of water-diluted 100 μM estradiol (E2) -/+ 200 μM DHPCC-9. **B.** Similar experiments were carried out also in the absence of E2. **C.** Pim expression levels were analysed by Western blotting from MCF-7 cells cultured in the presence of increasing concentrations of estradiol. β-actin staining was used as a loading control. **D.** Untransfected PC-3 cells were grown on the CAM for 5 days and treated daily with DMSO, 100-200 μM DHPCC-9 or 5 μg/ml of DAPT. A minimum of three separate experiments were performed. Shown are average tumor sizes from one representative experiment. **E.** N1ICD protein was stained from paraffin-embedded samples of orthotopic prostate xenografts that had been formed by mock-transfected PC-3 cells or cell stably overexpressing Pim1 or Pim3 [25]. Part of the mice with Pim-3-expressing xenografts had been daily treated with 50 mg/kg of DHPCC-9. After scanning, manual double-blinded analyses were performed to non-necrotic tissue sections. Shown are the average numbers of cells with nuclear Noth1 ICD. **F.** Representative images show cleaved Notch1 staining (brown).

Finally, we probed the possible synergistic effects of Pim and Notch activity on tumor growth. For this purpose, we used the well-established chick embryo chorioallantoic membrane (CAM) xenograft model [35], which we have previously used to assess the effects of Notch inhibition on breast and prostate cancer growth [36]. MCF-7 breast cancer cells transiently overexpressing N1ΔE wild-type or phosphomutants were xenografted onto CAM, and tumor growth was followed for 5 days. Part of the samples was treated with estradiol, which has been shown to upregulate Pim1 expression in the hormone-dependent MCF-7 cells [37]. In the presence of estradiol, wild-type Notch1 and the SE mutant both enhanced tumor growth, while the SA mutant strongly suppressed it (Figure 9A). Furthermore, the Pim inhibitor DHPCC-9 efficiently reduced Notch1-induced tumor growth to the same level as the SA mutation. In the absence of estradiol, the tumor-promoting effect of Notch1 was almost lost (Figure 9B). Intriguingly, already nanomolar concentrations of estradiol upregulated Pim1 and Pim3, but not Pim2 protein levels in cultured MCF-7 cells (Figure 9C), further supporting the conclusion that their expression is essential for full Notch1 activity.

We also examined the effects of Pim and Notch inhibitors on the growth of untransfected PC-3 cells on CAM. Pim inhibition by DHPCC-9 or blockade of Notch signaling by DAPT efficiently reduced tumor volume, while the most pronounced tumor-suppressive effects were obtained by a combinatorial treatment (Figure 9D). Furthermore, DAPT treatment was also able to decrease growth of tumors formed by PC-3 cells stably overexpressing Pim1 (Supplementary Figure 4).

**Figure 10:**
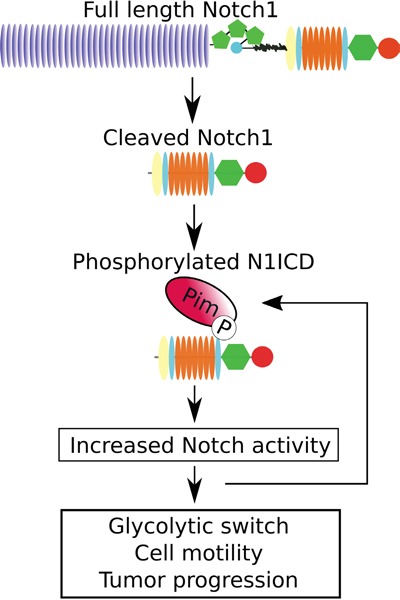
Schematic diagram of the effects of Pim kinases on Notch1 signalling

To further verify the cross-talk between Pim and Notch proteins in prostate cancer, we analysed the presence of nuclear Notch1 in orthotopic prostate xenografts. We have previously shown that Pim overexpression induces metastatic growth of PC-3-derived orthotopic xenografts in mice [25]. Immunostaining of the xenografted samples showed enhanced numbers of N1ICD-positive nuclei in tumors with stable Pim1 or Pim3 overexpression, while mock-transfected xenografts or Pim3-overexpressing xenografts from mice treated with the Pim inhibitor DHPCC-9 displayed fewer N1ICD-positive nuclei (Figure 9E-9F). This is in line with the observed changes in N1ICD localization in cultured PC-3 cells after Pim inhibition or overexpression of the phosphodeficient Notch1 mutant (Figure 3A-3B). These data together with the CAM results suggest that Pim/Notch crosstalk drives tumor progression (Figure 10), and that combinatorial therapies might be beneficial for cancer patients with deregulated expression of both Pim and Notch proteins.

## DISCUSSION

In this report, we have unraveled a novel link between Pim kinases and Notch1, which is relevant for the progression of both breast and prostate cancer. We show that all three Pim family kinases phosphorylate the intracellular domain of Notch1 (N1ICD), and thereby stimulate the nuclear localization and transcriptional activity of N1ICD. Pim kinases also phosphorylate Notch3, but not Notch2. Notch4 lacks Pim consensus sites and is thus not expected to be a Pim substrate. Here we focused on the phosphorylation of Notch1, as expression of Notch 3 was restricted to MCF-7 breast cancer cells and not present in PC-3 prostate cancer cells, and the exact role of Notch3 in tumor progression is still under debate [38, 39]. The Pim1 phosphorylation target site at S2152 resides in a linker between two clusters of basic amino acids, the entire sequence of which has been observed to contain a nuclear localization signal (NLS) for N1ICD [40]. N1ICD has been recognized as a target for the nuclear transport receptor importin-α, [41], and the linker sequence between the basic amino acid clusters has been proposed to facilitate direct association with importin-α [42, 43]. Our findings support this hypothesis and suggest that nuclear transport is associated with phosphorylation of S2152.

Pim-induced increase in the nuclear localization of N1ICD is observed in both cultured cells and in orthotopic prostate cancer xenografts, while Pim inhibitors or mutagenesis of the phosphorylation target site have opposite effects. The importance of our data is further enforced by the conservation of the Pim target site in Notch1 across several animal species [44]. Phosphorylation is not essential for the ability of Pim1 and Notch1 to interact with each other, as both wild-type Notch1 and phosphodeficient or phosphomimicking mutants all colocalize and physically interact with Pim1. This correlates with our previous observations demonstrating that both wild-type and kinase-deficient Pim1 can interact with Pim substrates [23, 45]. Our data indicate that endogenous Pim1 and Notch1 interact already in the cytoplasm, which supports the notion that Pim1 enhances nuclear localization and activity of Notch1.

Phosphorylation of Notch1 by Pim kinases promotes motility of prostate cancer cells, as demonstrated both by the phosphomutants and by the ability of either Pim or Notch inhibitors to block the pro-migratory effects of Notch or Pim proteins, respectively. In breast cancer cells, Pim-mediated phosphorylation of Notch1 balances cell metabolism, while its inhibition enforces glycolytic metabolism via defects in mitochondrial function, as previously shown for Notch inhibition [9]. This conclusion is supported by the reported abilities of Pim kinases to preserve mitochondrial integrity [27] and to regulate glycolysis and mitochondrial biogenesis by influencing expression of PGC-1α (peroxisome proliferator-activated receptor γ coactivator 1α) and c-Myc [28], which also is a transcriptional target for Notch1 [46]. Similar metabolic effects were not observed in prostate cancer cells, which are energetically less dependent on glycolysis [47].

The impact of Pim-mediated phosphorylation of Notch on tumor progression is demonstrated by our *in vivo* data from the CAM assays, where Pim and Notch synergistically enhance tumorigenic growth of both breast and prostate cancer cells. Treatment with the Pim inhibitor DHPCC-9 efficiently blocks the tumor-promoting effects of Notch1, and vice versa, the γ-secretase inhibitor DAPT abrogates the tumor-promoting effects of Pim1. Furthermore, we show that simultaneous inhibition of both Pim and Notch activities more efficiently inhibits tumor growth than targeting either one alone.

Since the Pim inhibitor DHPCC-9 and the S2152A mutation in Notch1 reduce Notch activity and tumor growth to a similar extent, this suggests that Pim kinases are the major kinases targeting Ser2152 of Notch1 in both MCF-7 and PC-3 cells. However, it remains possible that also other kinases such as Akt target it, since Akt is known to share some but not all substrates with Pim kinases [48]. Interestingly, we observed that up- or downregulation of Notch1 activity correlates with increased or decreased Pim expression levels, respectively. Thus, there may be a positive feedback loop, through which phosphorylation of Notch1 by Pim1 results in increased Pim levels to further enhance Notch1 activity, but not Notch1 expression.

Since Pim kinases and Notch1 play important functions in tumorigenesis, it is not surprising that there are major efforts underway to target their activities for cancer therapy [1, 19, 21]. We show that inhibition of Pim-mediated phosphorylation of Notch1 efficiently reduces tumor growth, and that simultaneous inhibition of Notch and Pim is even more effective. Our data suggest that the synergy between Pim and Notch leads to a more malignant behavior. Hence, combinatorial targeting of Pim and Notch proteins or their downstream targets may provide novel and effective approaches for cancer therapy.

## MATERIALS AND METHODS

### Cell culture and chemical compounds

Human PC-3 prostate cancer and MCF-7 breast cancer cell lines and their derivatives were cultured as previously described [9, 24, 25]. Stable control or human Pim1-overexpressing PC-3 cell lines were cultured in the presence of 200 μg/ml G418. For endogenous Notch activation, PC-3 cells were cultured on plates treated with 50 μg/ml of protein G/PBS overnight, 10 mg/ml BSA/PBS for 1 h and finally recombinant 2 μg/ml of Jagged1-FC or control FC as previously described [17]. To induce Notch1 expression in the stable MCF-7/N1ΔE cell line, 1 μg/ml of doxycycline (Sigma-Aldrich, St. Louis, MO, USA) was used. Pim kinase activity was inhibited by DHPCC-9 (1,10-dihydropyrrolo[2,3-a]carbazole-3-carbaldehyde; [24, 49]) or SGI-1776 (N-[(1-methylpiperidin-4-yl)methyl]-3-[3-(trifluoromethoxy)phenyl]imidazo[1,2-b]pyridazin-6-amine; S2198, SelleckChem, Houston, TX, USA), while the γ-secretase inhibitor DAPT (N-[N-(3,5-difluorophenacetyl)-L-alanyl]-S-phenylglycine t-butyl ester; Calbiochem, San Diego, CA, USA) was used to prevent Notch cleavage and nuclear entry. The inhibitors were diluted in DMSO, which was also used as a control (maximum concentration in cell culture 0.1%). Various concentrations of estradiol (E2) (E8875, Sigma-Aldrich) were used for treatment of cultured or xenografted MCF-7 cells. MTT (3-(4,5-dimethylthiazol-2-yl)-2,5-diphenyl tetrazolium bromide) (Sigma-Aldrich) was used to measure cell viability.

### DNA constructs and mutagenesis

Human *PIM* cDNAs were PCR-cloned from a human kinome cDNA collection [50] to the eukaryotic expression vector pcDNA™3.1/V5-His-C (Invitrogen, Carlsbad, CA, USA). Following primers were used for cloning: For 5′-C CCA AGC TTG ACC ATG CTC TTG TCC AAA ATC AAC-3′ and Rev 5′-CAG AAT TCC TTT GCT GGG CCC C-3′ (*PIM1*); For 5′-C CCA AGC TTG ACC ATG TTG ACC AAG CCT CTA CAG-3′ and Rev 5′-CAG AAT TCC GGG TAG CAA GGA CCA GG-3′ (*PIM2*); For 5′-C CCA AGC TTG ACC ATG CTG CTC TCC AAG TTC G-3′ and Rev 5′-CAG AAT TCC CAA GCT CTC GCT GCT GG-3′ (*PIM3*). Human *PIM1*, *PIM2* and murine *pim3* (a kind gift from A. MacDonald, University of Dundee, Dundee, UK) were further cloned to pGEX-6P-1 vector (GE Healthcare Life Sciences, Little Chalfont, UK) for GST fusion protein production. Following primers were used for cloning: For 5′-GCC GAA TTC ATG CTC TTG TCC-3′ and Rev 5′-GGG GTC GAC CTA TTT GCT GGG CC-3′ (*PIM1*); For 5′-GCC GGA TCC ATG TTG ACC AAG CC-3′ and Rev 5′-GGC GTC GAC TTA GGG TAG CAA GG-3′ (*PIM2*); For 5′-GCC GAA TTC ATG CTG CTG TCC-3′ and Rev 5′-GCC CTC GAG TCA CAA GCT CTC ACT GC-3′ (*pim3*). Human *PIM1* cDNA without a Stop codon and with a Kozak sequence (Genewiz Inc., South Plainfield, NJ, USA) was cloned into the pTagRFP-N vector (FP142; Evrogen, Moscow, Russia).

To prepare kinase-deficient (KD) human Pim mutants, the ATP-binding lysines were converted into methionines using the QuikChange site-directed mutagenesis kit (Stratagene, Agilent Technologies, Santa Clara, CA, USA). Following primers were used for mutagenesis: For 5′-GTG GCC ATC ATG CAC GTG GAG AAG GAC CGG ATT T-3′ and Rev 5′-CTC CAC GTG CAT GAT GGC CAC CGG CAA GTT G-3′ (Pim1 K67>M); For 5′-GTG GCC ATC ATG GTG ATT CCC CGG AAT CGT GTG-3′ and Rev 5′-GGG AAT CAC CAT GAT GGC CAC CTG GAG TCG ATC TG-3′ (Pim2 K61>M); For 5′-GTG GCT GTG ATG CAC GTG GTG AAG GAG CGG GT-3′ and Rev 5′-CAC CAC GTG CA TCA CAG CCA CCG GGA GCC C-3′ (Pim3 K69>M).

Following constructs were used for overexpression of murine Notch intracellular domains: N1ICD-pGEX-4T-3, N1ΔE-pCS2+ and GFP-N1ΔE [46]. N2ICD and N3ICD were cloned to pGEX-6P-3 by cutting the ICDs from the corresponding p3xFlag-CMV-7.0 constructs [51]. Site-directed mutagenesis of NICDs was performed by Stratagene Ultra Pfu DNA polymerase. Following primers were used for mutagenesis: For 5′-GCC ACA CAG GGA AAG AAG GCG CGC AAG CCA GC T ACC AAA GGG C-3′ and Rev 5′-G CCC TTT GGT AGC TGG CTT GCG CGC CTT CTT TCC CTG TGT GGC-3′ (Notch1 S2152>A, “SA”); For 5′-G GAC CTC AAG GCG CGC AGG AAG AAG GCA CAG GAT GGC AAG GGC-3′ and Rev 5′-GCC CTT GCC ATC CTG TGC CTT CTT CCT GCG CGC CTT GAG GTC C-3′ (Notch1 S2173>A); For 5′-CC ACA CAG GGA AAG AAG GCG CGC AAG CCC GAG ACC AAA GGG-3′ and Rev 5′-CCC TTT GGT CTC GGG CTT GCG CGC CTT CTT TCC CTG TGT GG-3′ (Notch1 S2152>E, “SE”).

### Transfections and transactivation assays

Fugene^®^ 6/HD (Promega, Fitchburg, Wisconsin, USA) 3:1 to DNA was used for PC-3 cell transfections, while MCF-7 cells were transfected either by jetPEI^®^ (Polyplus Transfection, New York, NY, USA) or by electroporation. For RNA interference, 200 nM non-targeting control siRNA or Pim-targeting siRNA oligonucleotides were transfected by Lipofectamine™ (Invitrogen/Life Technologies, Carlsbad, CA, USA). Following oligonucleotides were used: non-targeting MISSION^®^ siRNA Universal Negative Control #1, SIC001 (Sigma-Aldrich), D-001810-01-20 ON-TARGETplus Non-targeting siRNA #1, (Dharmacon, Lafayette, CO, USA), 5′-GAU GGG ACC CGA GUG UAU A-3′ J-003923-09-0020 ON-TARGETplus PIM1 siRNA (Dharmacon), 5′-GUG GAG UUG UCC AUC GUG ACA UU-3′ 5′-UGU CAC GAU GGA CAA CUC CAC UU-3′ PIM2 siRNA (Sigma-Aldrich), 5′-GGC GUG CUU CUC UAC GAU AUG UU-3′ 5′-CAU AUC GUA GAG AAG CAC GCC UU-3′ PIM3 siRNA (Sigma-Aldrich). Notch activity was measured by a 12xCSL luciferase reporter construct, while β-galactosidase was used for normalization as previously described [52]. Constitutively active CMV-luciferase construct (a kind gift from J. Ivaska, Turku Centre for Biotechnology, Turku, Finland) was used as an additional control.

### *In vitro* kinase assays, mass spectrometry and *in silico* analysis

GST-tagged fusion proteins were separated from glutathione sepharose beads by 30 mM glutathione in 75 mM Tris-HCl (pH 8.0), thereafter GST-tagged Pim and Notch family members were subjected to similar *in vitro* kinase reactions as previously described [53]. Following kinase assays, mouse N1ICD was subjected to in-gel trypsin digestion and TiO_2_ affinity chromatography as previously described [54]. Liquid chromatography-tandem mass spectrometry (LC-MS/MS) was performed using a Q Exactive mass spectrometer (Thermo Fisher Scientific). Database search was performed using Mascot 2.4 (Matrix Science) via Proteome Discoverer 1.3 (Thermo Fisher Scientific) against the Swiss-Prot (*E. coli* and recombinant proteins) database. Label-free quantification was performed using Progenesis LC-MS 4.0 (Nonlinear Dynamics). Protein sequence comparison was performed by BLAST (Basic Local Alignment Search Tool by The National Center for Biotechnology Information), while the sequences were obtained from Swiss-Prot Universal Protein Resource Knowledgebase.

### Fluorescence microscopy

PC-3 cells were plated on cover glasses and transiently transfected with RFP- or GFP-tagged expression vectors. After 48 hours, samples were fixed with 4% paraformaldehyde, washed with PBS and mounted with Mowiol. Samples were imaged by Leica TCS SP1 and TCS SP5 confocal microscopes with HCX PL APO CS 63×1.3 Oil objective with LCS 2.61 or LAS AF Application (Leica Microsystems CMS GmbH, Mannheim, Germany). Excitation wavelengths were 488 nm (GFP) and 561 nm (RFP), while emission wavelengths were 500-535 nm (GFP) and 599-651 nm (RFP). Sequential scanning was performed to reduce the background signal in colocalization imaging. Physical interactions between tagged proteins were measured by fluorescence-lifetime imaging microscopy (FLIM) as previously described [55]. By this method, fluorescence resonance energy transfer between two closely located fluorophores can be detected by measuring the change in the donor fluorophore (e.g. GFP) lifetime in the presence or absence of the acceptor (e.g. RFP) [56].

To analyse interactions between endogenously expressed Pim1 and Notch1 proteins, *in situ* proximity ligation assays (PLA) were carried out as previously described [57]. For this purpose, MCF-7 cell samples were fixed for 10 min with methanol and 1 min with acetone, and stained with Pim1 (ab117525, Abcam, Cambridge, UK), Notch1 (C20, Santa Cruz Biotechnology, Dallas, TX, USA) or CoxII (12C4, Santa Cruz) primary antibodies. Thereafter, the assays were continued using Duolink DUO92102 reagents (Sigma-Aldrich) according to manufacturers instructions. Finally, samples were mounted with DAPI-containing medium (Duolink DUO82040, Sigma-Aldrich), and imaged using the Zeiss LSM760 (ZEISS, Oberkochen, Germany) confocal microscope.

### Protein analyses

Western blotting samples were prepared and run as previously described [9]. Notch1 immunoprecipitation was performed with anti-cleaved Notch1 SAB4502019 antibody (Sigma-Aldrich) as previously described [17]. Primary antibodies from Cell Signaling Technology (Danvers, MA, USA) were diluted 1:1000 (anti-Pim1 #2907; anti-Pim2 #4730; anti-Pim3 #4165; anti-β-actin #4970S, anti-Notch1 Val1744, anti-phospho Ser/Thr (RXXS*/T*) #9614), while their secondary antibodies were diluted 1:5000 (anti-mouse #7076, anti-rabbit #7074). Other primary antibodies were diluted 1:500 (anti-Pim1 12H8, Santa Cruz Biotechnology) or 1:1000 (anti-Notch1 activated ab8925, Abcam; anti-HSC70/HSP73 1B5, Enzo Life Sciences, Farmingdale, NY, USA). Amersham™ ECL™ Plus/Prime reagents (GE Healthcare Life Sciences, Little Chalfont, UK) were used for chemiluminescence reactions. Signal intensities from SDS-PAGE and Western blotting samples were analysed by the ChemiDoc™ MP Imaging System with Image Lab software Version 4.0 (Bio-Rad Laboratories, Inc., Hercules, CA, USA). Relative signal intensities were calculated based on protein loadings or control stainings.

### Wound healing assays

A confluent PC-3 cell layer was scratched by a 10 μl pipette tip 24 h after transfection or Notch activation. Wounded cells were either left untreated or treated with DMSO-diluted compounds. Samples were imaged by Olympus CK40 microscope (Olympus Corporation Tokyo, Japan) with 20x enlargement and analySIS getIT 5.0 software (Olympus Soft Imaging Solutions GmbH, Münster, Germany), and analysed as previously described [24].

### Proliferation and viability assays

IncuCyte™ (Essen BioScience, Ann Arbor, MI, USA) was used for automatic measurement of cell confluency on 96-well plates. Cell viability was measured by MTT assays as previously described [24].

### Glucose uptake

MCF-7 cells were incubated with 100 μM fluorescent 2-NBDG (2-(*N*-(7-nitrobenz-2-oxa-1,3-diazol-4-yl)amino)-2-deoxyglucose), after which cells were detached, washed and analysed by flow cytometer BD FACSCalibur with BD CellQuest PRO v.5.1.1 software (BD Biosciences, San Jose, CA, USA) as previously described [9].

### Mitochondrial membrane potential

Flow cytometry was applied for analysis of mitochondrial permeability using the tetramethylrhodamine methyl ester (TMRM) dye as previously described [9].

### Lactate production

Conditioned medium was collected and deproteinated with 300 mM perchloric acid for 10 min on ice. After centrifugation, the supernatant was neutralized with 300 mM potassium hydroxide. After another centrifugation, lactate was converted to pyruvate by lactate dehydrogenase in the presence of nicotinamide adenine dinucleotide (NAD) (both from Sigma-Aldrich). Samples were incubated for 1 h at 37°C in a buffer containing 400 mM hydrazine sulphate and 500 mM glycine, after which formation of NADH was measured spectrophotometrically at 340 nm by Envision Multilabel Reader (PerkinElmer, Turku, Finland).

### Chorioallantoic membrane (CAM) model

Using the CAM model [35], 0.5-2 × 10^6^ cancer cells were transplanted onto the CAM of fertilized chicken eggs. The cells were allowed to form tumors and become infiltrated with the vasculature of the CAM for 5 days, during which tumors were treated daily with estradiol, DHPCC-9 or DAPT as indicated in the figures. The estradiol concentrations were chosen to be within the same range as previously used in mouse experiments [58]. On day 5, the tumors were fixed *in ovo* with 3% paraformaldehyde for 3h, removed, dehydrated and weighted.

### Immunohistochemistry

Samples for immunohistochemical analysis were gained from a previous study [25]. Animal experiment procedures were approved by the Provincial State Office of Western Finland with the licence ID ESAVI/3937/04.10.03/2011. Shortly, stable Pim1, Pim3, or empty plasmid (mock) overexpressing PC-3-derived prostate xenografts were allowed to grow for approximately three weeks, while part of the mice were daily treated with 50 mg/kg of the Pim inhibitor DHPCC-9. Antigen retrieval and peroxidase blocking were performed to paraffin-embedded tumor samples as previously described [9]. TBS was used instead of PBS. Samples were blocked in Dako Antibody Diluent (S0809, Agilent Technologies, Dako Denmark A/S, Glostrup, Denmark) for 10 min at RT, stained with primary antibody anti-Notch1 (Val1744, Cell Signaling Technology) 1:500 for 1 h at RT and secondary antibody Poly-HRP-Anti-rabbit IgG (DPVR55HRP, Agilent Technologies) for 30 min at RT. DAB treatment and hematoxylin counterstaining have been previously described [9]. Whole tumor scanning was performed as previously reported [25]. Double blind analysis were performed manually, and necrotic areas were left out from analysis.

### Statistical analysis and figure preparation

Bar graphs were produced by Microsoft Excel 2013 or GraphPad Prism 4.00 and results were analysed by Student's t-test and ANOVA. Pearson correlations for mRNA levels were obtained from the MediSapiens database (medisapiens.com). In each analysis, P<0.05 was used as a limit for significant difference. Error bars represent standard deviations. Figures were prepared by Corel Draw X5 or Adobe Illustrator CS5 15.0.0.

## SUPPLEMENTARY FIGURES AND TABLE


