# Supplementary material for: Phosphorylation of Notch1 by Pim kinases promotes oncogenic signaling in breast and prostate cancer cells
Source: Oncotarget. 2016 May 7;7(28):43220–38. doi: 10.18632/oncotarget.9215 (PMC5190019; doi:10.18632/oncotarget.9215)
Supplement: Supplementary file 1 [file oncotarget-07-43220-s001.pdf]

Phosphorylation of Notch1 by Pim kinases promotes oncogenic signaling in breast and prostate cancer cells

SUPPLEMENTARY FIGURES AND TABLE

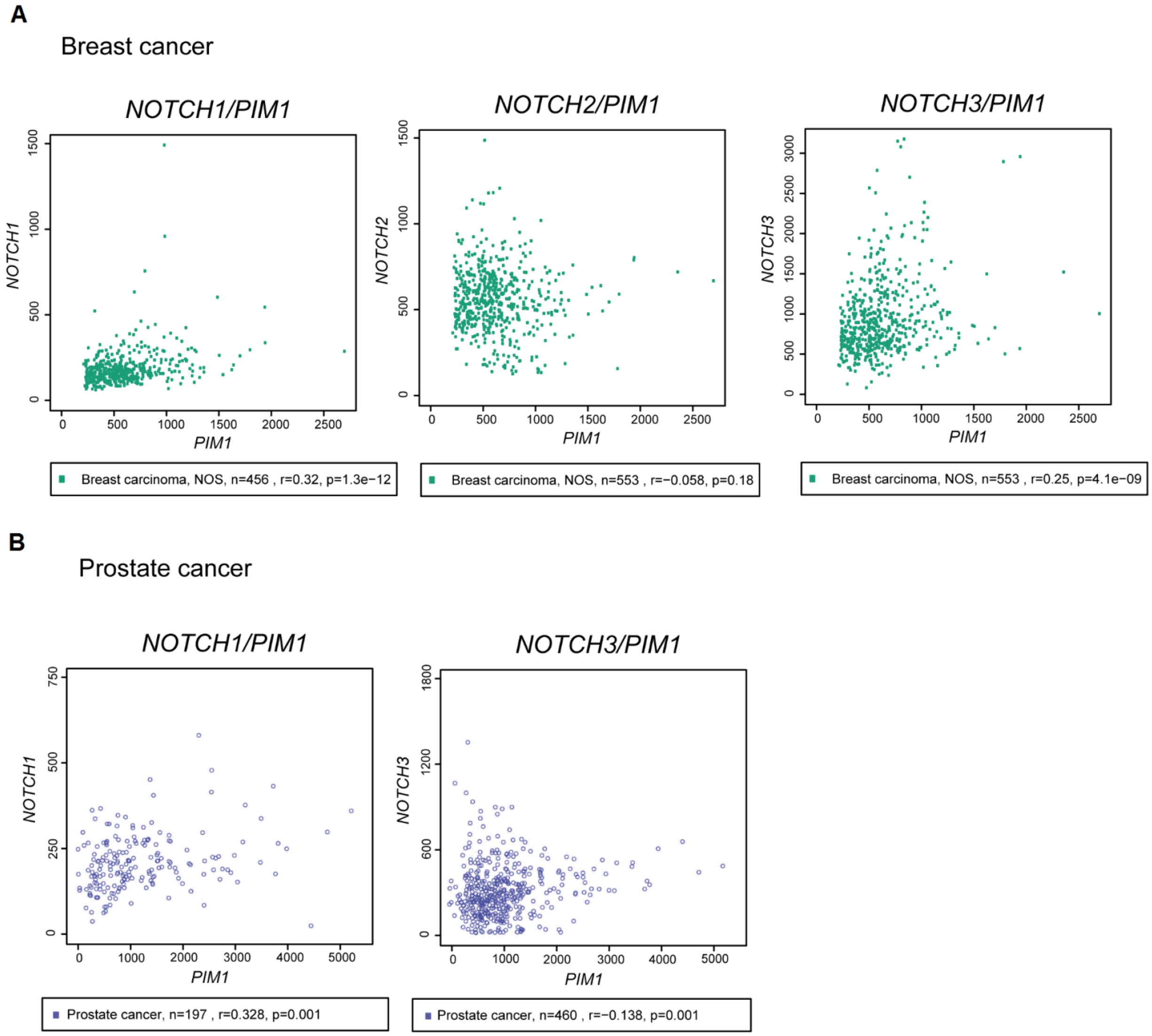

Supplementary Figure S1: *PIM1* expression correlates with *NOTCH1* and *NOTCH3* in breast and prostate cancer samples. Correlation of *PIM1* and *NOTCH* mRNA levels were determined from patient samples according to information from MediSapiens.com. **A.** *NOTCH1, NOTCH2*, *NOTCH3* and *PIM1* expression were found in breast cancer samples. **B.** *NOTCH1*, *NOTCH3* and *PIM1* expression were found in prostate cancer samples.

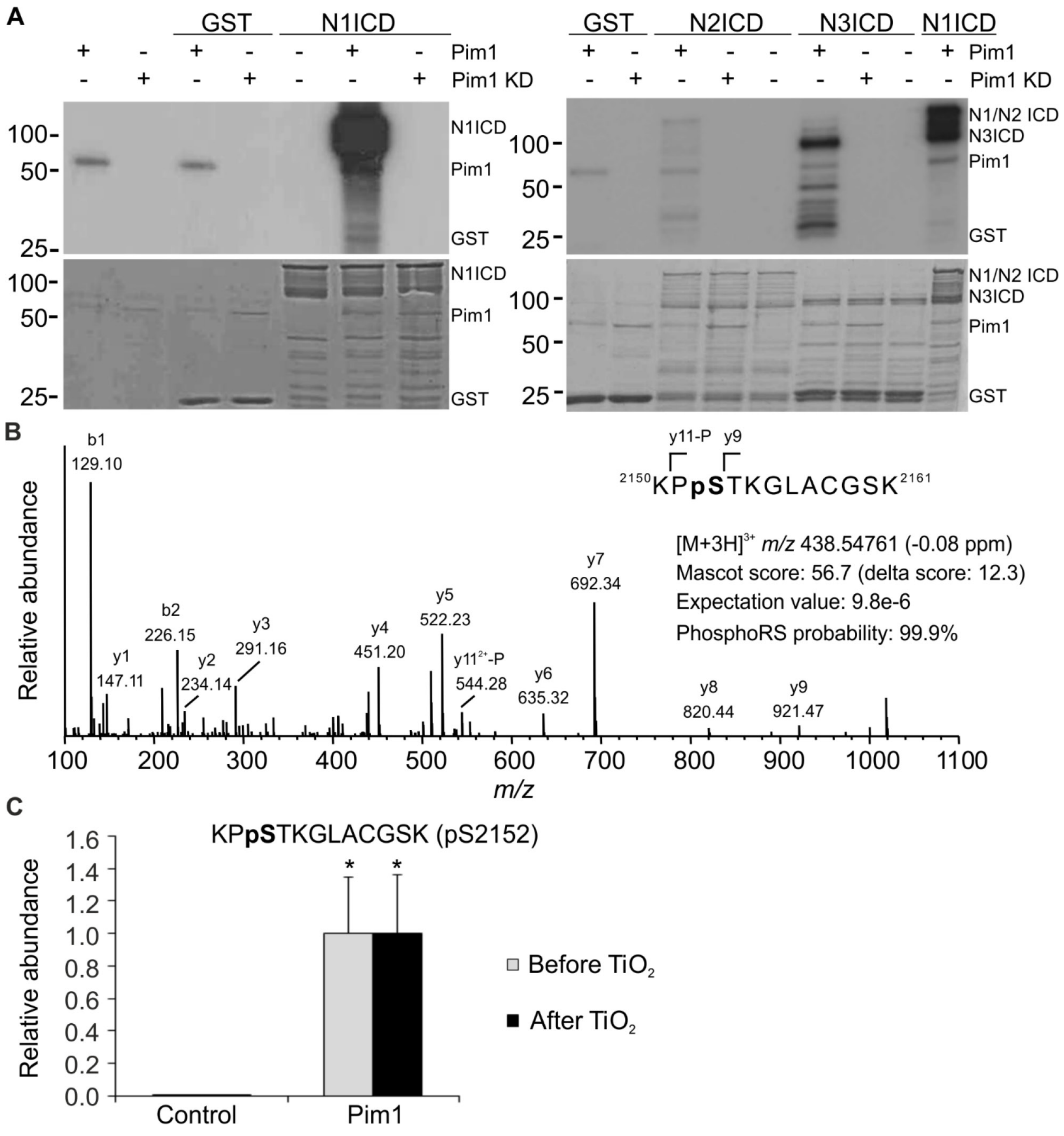

Supplementary Figure S2: Notch1 is phosphorylated by Pim1 at serine 2152. GST-tagged Pim1, its kinase-deficient (KD) mutant and Notch (N) intracellular domains were incubated in *in vitro* kinase reactions. **A.** Shown are autoradiograms (above) and protein staining (below). **B.** Tryptic digestion and TiO_2 phosphopeptide enrichment followed by mass spectrometry revealed S2152 phosphorylation and C2158 carbamidomethylation in N1ICD. **C.** Shown is label-free phosphopeptide quantification normalized to N1ICD abundance (n=3).

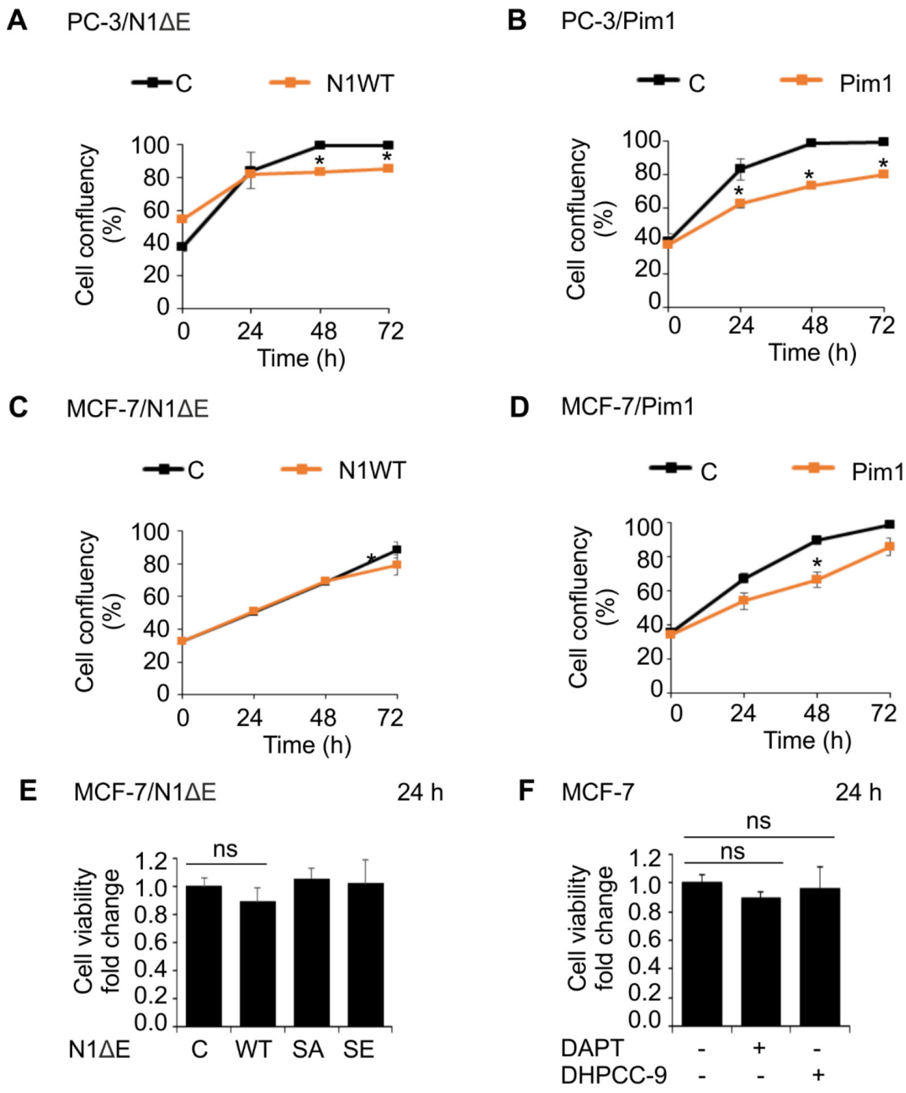

Supplementary Figure S3: Notch and Pim activity in PC-3 and MCF-7 cell proliferation or viability. Cells were transiently transfected with N1ΔE wild-type (WT) or phosphomutants (SA and SE) or Pim1. **A.** Confluency of N1ΔE-overexpressing PC-3 cells. **B.** Confluency of Pim1-overexpressing PC-3 cells. **C.** Confluency of N1ΔE-overexpressing MCF-7 cells. **D.** Confluency of Pim1-overexpressing MCF-7 cells. Various cell concentrations were tested with similar results and shown are average results with one concentration and parallel samples. **E.** Cell viability was analysed in MCF-7 cells by MTT assays after N1ΔE overexpression. **F.** Untransfected MCF-7 cells were also treated with 5 µg/ml of DAPT or 10 µM DHPCC-9, after which MTT assays were performed. Shown are combined results from at least two independent experiments. One day after transfection was considered as the 0 h time-point.

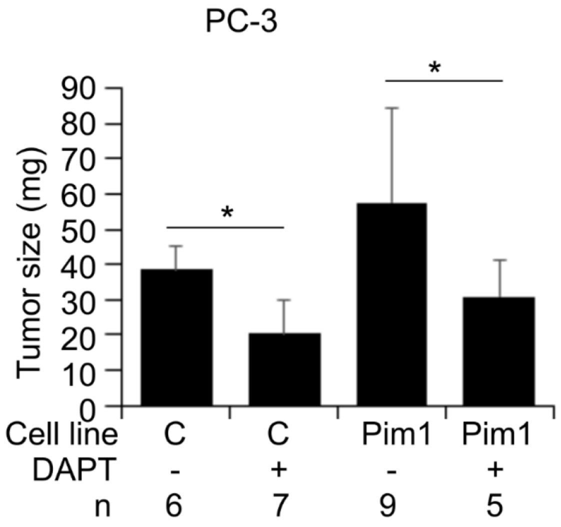

Supplementary Figure S4: DAPT decreases Pim1-induced PC-3 tumor growth on CAM. Stable control (C) or Pim1-overexpressing (Pim1) PC-3 cells were grown on the chorioallantoic membranes (CAM) of chicken embryos. Tumors were treated for 5 days with 5 µg/ml of DAPT or they were control-treated with equal amounts of DMSO. Shown are sample numbers (n) and average tumor sizes from one experiment.

Supplementary Table S1: Pim target sites are highly conserved in Notch receptors

Amino acids in comparison	Amino acid sequence comparison	Accession number	Notch member	Species
2145-2157	GKKARK--PSTK---GLA	Q01705	Notch1	mouse
2155-2167	GKKVRK--PSSK---GLA	P46531	Notch1	human
2098-2115	GKKARRPNTKSTMPTSLP	O35516	Notch2	mouse
2100-2117	GKKSRRPSAKSTMPTSLP	Q04721	Notch2	human
2060-2074	GTKKSRRPPGKT---GLG	Q61982	Notch3	mouse
2059-2073	GSKKSRRPPGKA---GLG	Q9UM47	Notch3	human
1834-1848	GGAAARC---RTLSAGAR	P31695	Notch4	mouse
1855-1869	GGALPRC---RTLSAGAG	Q99466	Notch4	human

Shown are sequence homologies between different Notch family members around Pim target site S2152 in mouse Notch1. Sequence data is received from UniProtKB/Swiss-Prot.
